# Multi-Enzymatic Synthesis of Lactobionic Acid Using Wood-Degrading Enzymes Produced by White Rot Fungi

**DOI:** 10.3390/metabo13040469

**Published:** 2023-03-24

**Authors:** Wiktoria Piątek-Gołda, Justyna Sulej, Marcin Grąz, Piotr Waśko, Ewa Janik-Zabrotowicz, Monika Osińska-Jaroszuk

**Affiliations:** 1 Department of Biochemistry and Biotechnology, Institute of Biological Sciences, Maria Curie-Sklodowska University, 19 Akademicka St., 20-033 Lublin, Poland; 2 Department of Plant Physiology and Biophysics, Institute of Biological Sciences, Maria Curie-Sklodowska University, 19 Akademicka St., 20-033 Lublin, Poland; 3 Core Facility of Biospectroscopy, Institute of Biological Sciences, Maria Curie-Sklodowska University, 19 Akademicka St., 20-033 Lublin, Poland; 4 Department of Cell Biology, Institute of Biological Sciences, Maria Curie-Sklodowska University, 19 Akademicka St., 20-033 Lublin, Poland

**Keywords:** oxidoreductive enzymes, lactobionic acid, enzymatic oxidation, secondary metabolite

## Abstract

Enzymes produced by white rot fungi are involved in the synthesis of secondary metabolites with valuable biotechnological properties. One of these metabolites is lactobionic acid (LBA). The aim of this study was to characterize a novel enzyme system consisting of a cellobiose dehydrogenase from *Phlebia lindtneri* (PlCDH), a laccase from *Cerrena unicolor* (CuLAC), a redox mediator (ABTS or DCPIP), and lactose as a substrate. We used quantitative (HPLC) and qualitative methods (TLC, FTIR) to characterise the obtained LBA. The free radical scavenging effect of the synthesised LBA was assessed with the DPPH method. Bactericidal properties were tested against Gram-negative and Gram-positive bacteria. We obtained LBA in all the systems tested; however, the study showed that the temperature of 50 °C with the addition of ABTS was the most advantageous condition for the synthesis of lactobionic acid. A mixture with 13 mM LBA synthesised at 50 °C with DCPIP showed the best antioxidant properties (40% higher compared with the commercial reagent). Furthermore, LBA had an inhibitory effect on all the bacteria tested, but the effect was better against Gram-negative bacteria with growth inhibition no lower than 70%. Summarizing the obtained data, lactobionic acid derived in a multienzymatic system is a compound with great biotechnological potential.

## 1. Introduction

Fungi are important agents in combating various fungal pests and plant diseases found in greenhouses, fields, and even post-harvest conditions [1]. They allow plants to grow more efficiently and protect them from pathogens; however, they also have a damaging effect on trees by breaking down the lignocellulosic biomass. The plant-fungus interaction helps to maintain the balance in nature. The favourable effects of fungi on plants mainly consist in cooperation with plants in a process called mycorrhiza [2]. This type of symbiosis involves mutual benefits. The fungi support the plant in taking up nutrients from the soil and the plant provides the fungi with carbohydrates [3]. However, fungi can also have a negative effect on plants. They attack injured or freshly felled trees, damage plant tissues, and may consequently even cause plant death. One of the plant diseases caused by fungi is white wood rot. The tree lignocellulosic biomass, which consists of a complex composed of carbohydrate polymers such as cellulose (40–60%), hemicellulose (20–40%), and lignin (10–24%), is degraded [4,5]. The tree quickly loses weight, begins to bow under pressure, and its wood becomes soft. This disease affects mainly broadleaf trees [6].

One of the fungi known to be responsible for white wood rot is *Cerrena unicolor*. It is a Basidiomycota fungus belonging to the family of *Polyporaceae* [7]. This fungus parasitizes living trees but also lives as a saprobe on dead wood. *C. unicolor* infects trees all year round and can attack several deciduous species but is also adept at decomposing dead conifer logs. In addition, the species *C. unicolor* is known as an enzyme producer, which is widely confirmed in the literature [8,9,10,11,12].

Another wood-decaying Basidiomycota fungus is *Phlebia lindtneri* belonging to the family *Meruliaceae*. This fungus species is relatively rare in nature, and its natural environment is boreonemoral forests [13]. It is a source of biotechnologically important biocatalysts such as laccase, cellobiose dehydrogenase, and protease [14,15,16]. Through the interaction of fungi with plants, fungi are able to secrete secondary metabolites that are used to degrade lignocellulosic biomass. It is well known that there are complex enzyme systems involved in the degradation of lignin; however, the enzymes used in this process have found other applications as well [17,18]. One of the fungal enzymes involved in the synthesis of secondary metabolites is cellobiose dehydrogenase (CDH). It is an extracellular hemoflavoenzyme classified into the class of oxidoreductases. It is secreted by many fungal species, including *Phlebia lindtneri*, which are responsible for wood degradation, and by phytopathogenic fungi [19]. With its unique structure and properties (i.e., antioxidant and antimicrobial effects), CDH exhibits great biotechnological and biomedical application potential [20,21,22,23]. Another important lignin metabolism-related enzyme is laccase (LAC). This belongs to a minor group of enzymes known as blue multicopper oxidases [24]. These enzymes are involved in the metabolism of a wide range of phenolic compounds, including polymeric lignin and humic substances [25]. Moreover, laccase can be used in the textile, paper, food, cosmetic, and pharmaceutical industries, in environmental protection, and in leather tanning [26,27,28,29,30]. Enzymes secreted by white rot fungi, such as cellobiose dehydrogenase and lactase, can be successfully used in the lactose oxidation reaction in which lactobionic acid (LBA) is produced. LBA is an oligoaldonic acid composed of galactose bound to a gluconic acid molecule [31]. LBA has great application potential and can be used in many industries. Due to its stabilising, moisturising, antioxidant, or antimicrobial properties, it has found a wide range of applications in cosmetology, the food industry—including the dairy industry—and in medicine as a drug and gene carrier [31,32,33]. At the same time, lactobionic acid is a secondary metabolite of some microbial species (i.e., *Acinetobacter halotolerans*, *Pseudomonas taetrolens*, *Enterobacter cloacae*); for this reason, we cannot exclude the possibility that the reactions shown in our laboratory studies may be analogous to those occurring in the environment [34,35,36].

The aim of this study was to obtain lactobionic acid using fungal enzymes whose main function is to degrade the lignocellulosic biomass of trees. The development of a multi-enzymatic system for the synthesis of LBA and determination of its antioxidant and antibacterial properties has allowed us to evaluate the biotechnological potential of this compound. The experimental plan carried out in this work involved the following steps: (a) the development of a multienzymatic system for the synthesis of lactobionic acid, (b) the qualitative and quantitative analysis of the lactobionic acid formed in the reaction, and (c) the determination of the antibacterial and antioxidant properties of LBA. The enzymes used in the study were isolated from the wood-degrading fungi *Phlebia lindtneri* (CDH) and *Cerrena unicolor* (LAC).

## 2. Materials and Methods

### 2.1. Materials and Microorganisms

All chemicals used in the study were of the highest available purity and analytical grade (≥98%). The media components and all other chemicals were purchased from Sigma Aldrich (Steinheim, Germany), Merck (Darmstadt, Germany), VWR (Vienna, Austria), Bio-Rad (Warsaw, Poland), or BioMaxima (Lublin, Poland). Deionised water was used to prepare aqueous solutions during the experiments.

Fungi *Phlebia lindtneri* (FCL22) producing cellobiose dehydrogenase were obtained from the culture collection of the Agriculture Academy in Cracow. Laccase produced by the white rot fungi *Cerrena unicolor* (FCL139) was obtained from the culture collection of Regensburg University in Germany. Both strains were deposited in the fungal collection at the Department of Biochemistry and Biotechnology at Maria Curie-Sklodowska University (Poland). The fungi were genetically identified, and their nucleotide sequences were deposited in GenBank with accession numbers DQ056858 (FCL139) and FJ594063 (FCL22).

The Gram-negative bacteria *Escherichia coli* ATCC 25922 and *Pseudomonas aeruginosa* ATCC 27853 and the Gram-positive bacteria *Staphylococcus aureus* ATCC 25923 and *Staphylococcus epidermidis* ATCC 14990 used in the experiments were obtained from the culture collection of the Department of Biochemistry and Biotechnology at Maria Curie-Sklodowska University (Poland). Due to the screening nature of the antibacterial assays, commonly used bacterial reference strains were selected for the study.

### 2.2. Culture Conditions and Purification of CDH and LAC

The white rot fungi *P. lindtneri* (FCL22) and *C. unicolor* (FCL139) were produced and isolated as described in previously published papers with modifications [11,19,21]. The culture supernatants (6000 mL) were collected and clarified by centrifugation at 4 °C for 30 min at 8000× *g* on a 6K15 apparatus (Sigma, Osterode am Harz, Germany). The samples were concentrated by ultrafiltration using a Prep/Scale TFF Cartridge ultrafiltration cell with a 10-kDa cut-off polyethylene sulfone membrane (PTGC, 0.09 m^2^) (Millipore, Bedford, MA, USA) and used as a source of enzymes to perform purification. The protein concentrate from *P. lindtneri* (source of CDH) was fractionated in ammonium sulphate at 30–50% saturation at 0 °C. The resulting protein pellet was dissolved in deionised water and desalted by diafiltration through centrifugal concentrators (Vivaspin Turbo 15) in polyethylene sulfone (PES) with a cut-off of 30 kDa (Sartorius, Göttingen, Germany). The purification of the enzymes (laccase and CDH) was performed using the AKTA-Prime purification system (GE Healthcare, Uppsala, Sweden) operating at 24 °C. The filtered sample was applied to a DEAE-Sepharose (fast flow) column (GE Healthcare, Uppsala, Sweden) previously equilibrated with 50 mM sodium acetate buffer (pH 5.0). Elution was performed using a linear NaCl gradient from 0 to 0.5 M in the same buffer at a flow rate of 3 mL/min. The fraction containing cellobiose dehydrogenase activity (PlCDH) was merged and concentrated using a PES Vivaspin Turbo 15 filter (Sartorius, Göttingen, Germany) with a cut-off of 30 kDa. The fraction containing laccase activity (CuLAC) was desalted and concentrated using a PES Vivaspin Turbo 15 filter (Sartorius, Göttingen, Germany) with a 10 kDa cut-off. Purified proteins were stored at −20 °C until further use.

### 2.3. Enzyme Activity Assay and Protein Determination

Cellobiose dehydrogenase activity was determined with the method proposed by Baminger et al. (1999) with modifications. PlCDH activity was measured in the presence of 2,6-dichloroindophenol (DCPIP) as an electron acceptor and lactose as a substrate (Sigma Chemical Co., St. Louis, MO, USA). The increase in absorbance was measured for 60 s at λ = 520 nm at 30 °C [20,37].

Laccase activity was determined using the method developed by Grzywnowicz and Leonowicz, (1981) with modifications. CuLAC activity was measured in the presence of 0.5 mM syringaldazine (4-hydroxy-3,5-dimethoxybenzaldehyde azine) (Aldrich, Saint Louise, MO, USA) in 0.1 mM citrate-phosphate buffer, pH 5.3. The increase in absorbance was measured for 60 s at λ = 525 nm at 25 °C [38].

The protein concentration was determined using the Bradford method with bovine serum albumin (BSA) as a standard [39].

### 2.4. Enzymatic Oxidation of Lactose and Synthesis of Lactobionic Acid (LBA)

**Figure 1:**
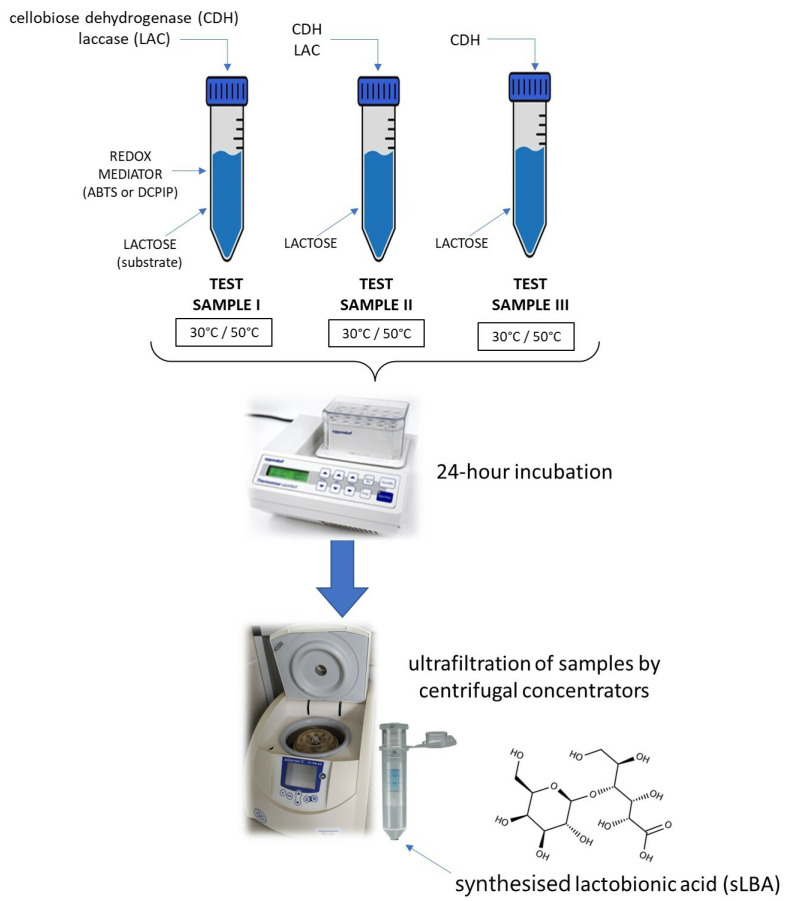
Schematic of the enzymatic reaction resulting in the formation of a mixture containing synthesised lactobionic acid (sLBA).

Several combinations of enzymatic lactose oxidation reactions were prepared to produce LBA (Figure 1).

The multi-enzymatic system consisted of the PlCDH and CuLAC enzymes in a 1:2 ratio, a 100 mM lactose substrate, and a mediator (ABTS or DCPIP). Furthermore, an experiment was performed using the PlCDH enzyme only with the substrate in a 1:1 ratio, as well as a sample with the two enzymes and lactose without the addition of the redox mediator. All assays were performed in two temperature conditions: 30 °C and 50 °C. After the incubations in different time intervals (0.5 h, 1 h, 3 h, 6 h, 8 h, and 24 h), the samples were ultrafiltrated through centrifugal concentrators (Vivaspin 500) in a polyethylene sulfone (PES) membrane with a cut-off of 10 kDa (Sartorius, Göttingen, Germany). Disaccharides and lactobionic acid present in the low molecular weight fraction were analysed [18,40,41].

#### 2.4.1. Determination of LBA Using High-Performance Liquid Chromatography

The concentration of lactobionic acid (LBA) and lactose was quantified using high-performance liquid chromatography (HPLC, Agilent Infinity 1260 equipped with RID and DAD detectors). An HPLC system equipped with a Bio-Rad Aminex HPX-87H column was operated at 50 °C with 0.45 mM H_2_SO_4_ as a mobile phase at a flow rate of 0.7 mL/min and an injection time set at 20 s [20].

#### 2.4.2. Determination of LBA Using Thin-Layer Chromatography

Thin-layer liquid chromatography (TLC) was carried out with the method proposed by Kiryu with modifications for qualitative determination of LBA in the samples. For this purpose, we used Kaisel Gel 60 TLC plates (Merck, Darmstadt, Germany) and the solvent system with ethyl acetate: 80% acetic acid: distilled water (3:2:1, by volume). After separation in the chamber, the plate was sprayed with 50% (*v*/*v*) H_2_SO_4_ in methanol and heated to 150 °C to reveal spots indicating the presence of lactose and lactobionic acid [42].

#### 2.4.3. Determination of LBA using Fourier-Transform Infrared Spectroscopy (FTIR) 

Infrared absorption spectroscopy measurements were performed with use of a Vertex 70 FTIR spectrometer (Bruker, Billerica, MA, USA). Spectra were recorded in the attenuated total reflection (ATR) configuration with a ZnSe crystal plate (45° cut, 10 internal reflections), with a resolution of data point per 2 cm^−1^ and a range between 4000 and 600 cm^−1^. Each spectrum was collected at room temperature as 32 scans, which were Fourier-transformed and averaged. The measurement chamber was purged with dry N_2_ gas during the experiments and for 30 min before the analyses. Pure organic solvents (Merck) were used to clean the ATR crystal plate before each experiment. All samples were deposited on the ATR crystal as a film, dried under a N_2_ flux for 15 min, and placed in a vacuum for at least 15 min. The Grams/AI software (ThermoGalactic Industries, Waltham, MA, USA) was used for spectra analysis. For easy comparison, all spectra were area normalised.

### 2.5. Antibacterial Activity Assay of LBA 

The antibacterial activity of LBA was measured using Gram-negative bacteria *E. coli* ATCC 25922 and *P. aeruginosa* ATCC 27853 and Gram-positive bacteria *S. aureus* ATCC 25923 and *S. epidermidis* ATCC 14990. Microdilution tests in broth were performed in 96-well microtiter plates. Mueller–Hinton broth medium (MH) (100 µL) was pipetted into each well, and then the enzymatically produced LBA (100 µL) was added. Next, the wells were inoculated with 10^4^ CFU/mL of the tested bacterial strains. MH medium samples from each experiment were tested for bacterial growth by measuring optical density (OD) on an Infinite 200 Pro microplate reader (Tecan, Männedorf, Switzerland) at a wavelength of 600 nm. The plates were incubated at 37 °C for 24 h, after which the absorbance measurement was repeated to determine the percentage inhibition of bacterial growth [20].

The results were given in a percentage of bacterial inhibition (IC%) according to the equation below:

*bacterial inhibition percentage (IC%)* = 100 − *(OD600assay/OD600positive control)* × 100

where OD600 is the optical density of the sample measured at 600 nm.

The spectrophotometric method with 2,3,5-triphenyltetrazolium chloride (TTC) solution developed by Veiga et al. (2019) with modifications was employed to determine the viability of the tested microorganisms. After 18 h of incubation, 1% TTC was added to each well and incubated at 37 °C for 30 min. After this time, the absorbance at 540 nm was measured [43]. The minimum inhibitory concentration (MIC) for cLBA was determined based on Fan’s method with modifications. Appropriate dilutions of lactobionic acid were prepared using Mueller–Hinton broth (MHB). The final cLBA concentrations were in the range of 0.5 to 35 mg/mL. The prepared cLBA solutions were added to the designated wells of a 96-well plate. Suspensions of the bacterial strains tested (*E. coli*, *S. aureus*, *S. epidermidis*, and *P. aeruginosa*) at a final concentration of 10^4^ CFU/mL were added to the respective wells. The plates were then incubated at 37 °C for 24 h, after which the MIC was determined using spectrophotometric measurements at 600 nm on an Infinite 200 Pro microplate reader (Tecan, Männedorf, Switzerland) [44].

### 2.6. Antioxidant Properties of LBA

The antioxidant properties of lactobionic acid were examined using the 2,2-diphenyl-1-picryl-hydrazyl-hydrate (DPPH) assay according to the spectrophotometric procedure described by Paduch et al. (2008) with some modifications. The standards (Trolox and Vitamin C), both well known for their strong antioxidant activity, were used as a positive control. Absorbance was measured spectrophotometrically at 515 nm with a microplate reader (Tecan, Männedorf, Switzerland) after 0.5 h, 1 h, 2 h, 3 h, 4 h, 5 h, and 6 h of incubation at room temperature. All measurements were carried out in triplicate. The percentage of DPPH oxidation reduction was calculated according to the formula:

DPPH scavenging effect(%) = [(X_0_ − X_1_)/X_0_] × 100

where X_0_ is the absorbance of the control sample and X_1_ is the absorbance of the standards or the tested compounds [21,45].

### 2.7. Statistical Analysis

The presented results are expressed as mean ± SD from three independent experiments (*n* = 3). The mean values and standard deviation were calculated using one-way ANOVA (Statgraphics Online) and then the means were compared using Tukey’s multiple range test. Excel program (Microsoft Office 2010 package) was used for the calculation of the data. Values of *p* ≤ 0.05 were considered statistically significant.

## 3. Results and Discussion 

### 3.1. Enzymatic Oxidation of Lactose and Synthesis of Lactobionic Acid (LBA)

**Figure 2:**
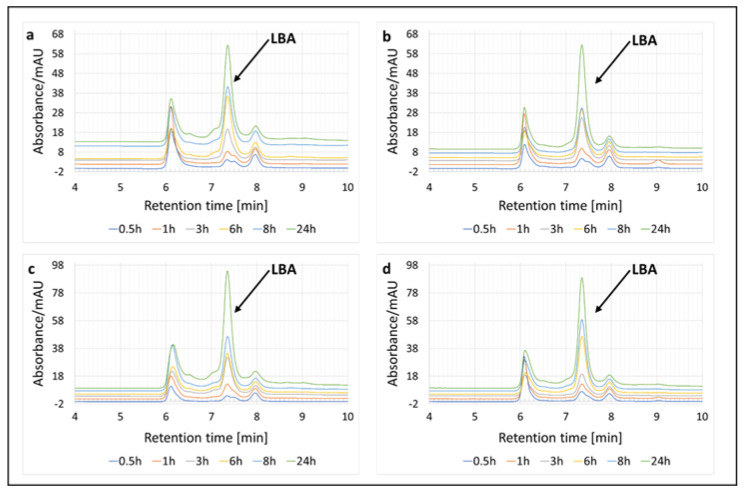
Enzymatic oxidation of lactose and qualitative analysis of the resulting product (lactobionic acid) using HPLC chromatography at 0.5 h, 1 h, 3 h, 6 h, 8 h, and 24 h. (**a**) Enzymatic system of PlCDH and lactose as a substrate at 30 °C. (**b**) Enzymatic system of PlCDH, CuLAC, and lactose as a substrate at 30 °C. (**c**) Enzymatic system of PlCDH and lactose as a substrate at 50 °C. (**d**) Enzymatic system of PlCDH, CuLAC, and lactose as a substrate at 50 °C.

Two enzyme systems were tested first, one containing the cellobiose dehydrogenase (PlCDH) enzyme and lactose and the other containing two enzymes (PlCDH and laccase (CuLAC)) and lactose. We based this experiment on our previous study, but made some modifications [20]. Originally, we wanted to determine the effect of incubation time on the amount of LBA produced. The enzyme systems were tested at two temperatures of 30 °C and 50 °C. The incubation was carried out for 24 h and samples were taken for analysis after 0.5 h, 1 h, 3 h, 6 h, 8 h, and 24 h. Using HPLC chromatography, the presence of lactobionic acid in the tested sample was confirmed (Figure 2).

**Figure 3:**
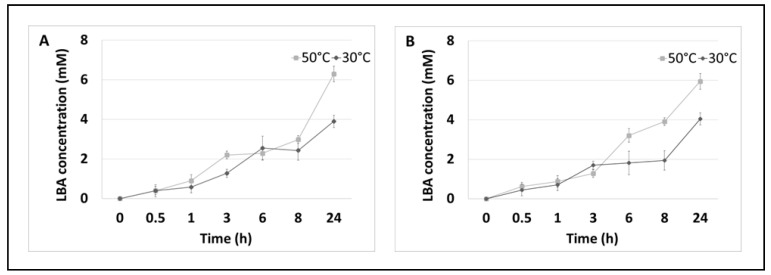
Enzymatic oxidation of lactose. (**A**) Enzymatic system of PlCDH and lactose as a substrate in two temperature conditions over 24 h. (**B**) Enzymatic system of PlCDH, CuLAC, and lactose as a substrate in two temperature conditions at 24 h. Error bars represent the standard deviation of the three experiments.

Moreover, quantitative analysis of the LBA formed in the reaction was also carried out by HPLC chromatography at different incubation times under two temperature conditions (Figure 3).

The results obtained in this assay confirmed that the multi-enzymatic reaction leads to the synthesis of LBA and that the acid concentration depends on the incubation time and temperature. The highest concentration (6.29 mM), which has a 13% conversion efficiency for lactose to LBA during the enzymatic oxidation of lactose, was observed after 24 h of incubation at 50 °C, and the type of the reaction substrate did not significantly affect the amount of LBA synthesised.

As shown in Figure 3, the concentration of LBA started to increase rapidly at 8 h of incubation. In both enzyme systems, the maximum conversion efficiency relative to the initial substrate concentration (lactose) was about 8% LBA at 30 °C and about 12% LBA at 50 °C. The highest concentration of sLBA was recorded after 24 h and was about 4 mM at 30 °C and about 6 mM at 50 °C regardless of the systems tested. 

In the next step, we decided to use the electron mediators in the enzyme systems, ABTS and DCPIP, to intensify the efficiency of the lactose oxidation process.

**Figure 4:**
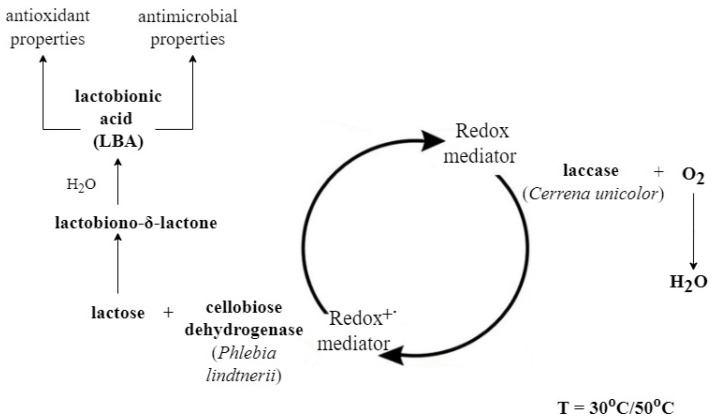
Scheme of the lactobionic acid synthesis process performed using the cellobiose dehydrogenase/lactose system in the presence of a redox mediator (ABTS or DCPIP). The reaction was carried out in two temperature conditions in parallel: 30 °C and 50 °C. PlCDH is able to catalyse the oxidation reaction of disaccharides linked by β-1-4-glycosidic bonds such as lactose. As a result of this process, lactobiono-δ-lactone is formed and spontaneously hydrolysed in an aqueous solution to aldonic acid (lactobionic acid) [20]. The reaction is catalysed by a redox mediator; when reduced in a lactose oxidation reaction, it can return to its oxidised form thanks to the presence of CuLAC. With the use of redox mediators, this process can proceed continuously [17].

The multi-enzymatic reaction used for the synthesis of lactobionic acid is schematically shown in the figure below (Figure 4). Once the LBA was obtained and chromatographically identified (TLC and HPLC analysis), we tested the applicability of the preparation in the biotechnology industry. For this purpose, we tested the antioxidant and antibacterial properties of the mixture containing sLBA. 

An enzyme system containing PlCDH, CuLAC, lactose and one of the two studied redox mediators (ABTS or DCPIP) was tested. The samples were checked at two temperatures, 30 °C and 50 °C, and incubation was carried out for 24 h.

**Figure 5:**
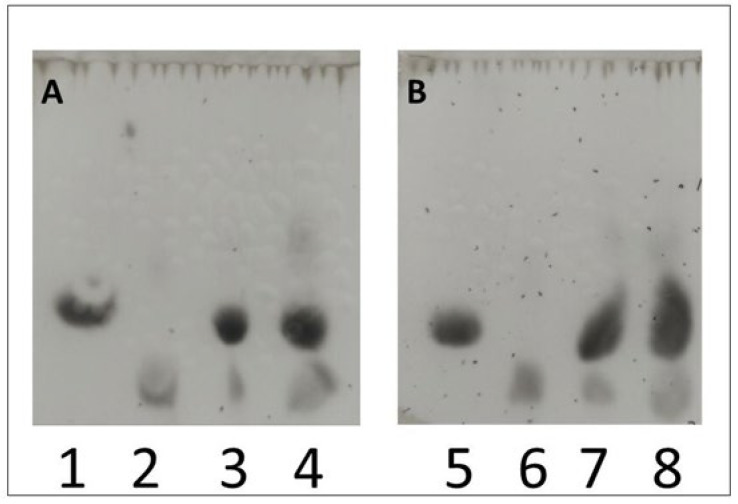
Qualitative study of lactobionic acid using the TLC method. Samples containing a redox mediator (ABTS (**A**) or DCPIP (**B**)) incubated at two different temperatures (30 °C or 50 °C) were used for the analysis. Lane 3 (30 °C) and 4 (50 °C) were samples with ABTS (**A**), while lane 7 (30 °C) and 8 (50 °C) represented those with DCPIP (**B**). Control samples were cLBA at a concentration of 20 mM (lanes 2 and 6) and 25 mM lactose in acetate buffer (lanes 1 and 5). After the stain analysis, the presence of both lactose (the oxidized substrate) and lactobionic acid (the product of the enzymatic reaction) was confirmed in the control samples.

The qualitative analysis of the resulting lactobionic acid was carried out using TLC (Figure 5). The use of this method allowed additional confirmation of the presence of synthesised LBA in the samples.

**Figure 6:**
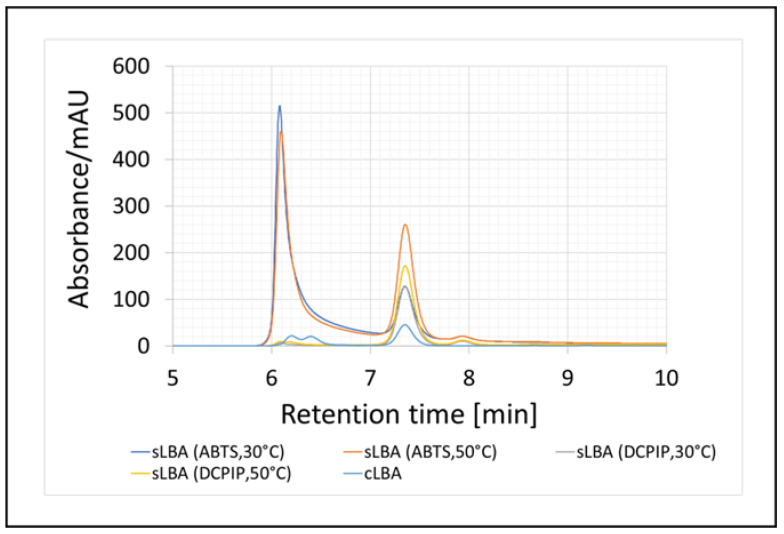
Enzymatic oxidation of lactose and qualitative analysis of the resulting product (lactobionic acid) using HPLC chromatography after 24-h enzymatic reaction. The synthesised acid was labelled as sLBA, while the commercial acid was labelled as cLBA. The reaction conditions are shown in brackets.

A chromatogram confirming the presence of sLBA in the redox mediated samples, for which we used commercial lactobionic acid (cLBA) as a standard, is shown below (Figure 6).

Moreover, using HPLC, we determined the amount of LBA formed in the enzymatic systems that we tested (Table 1).

It was observed that the temperature of the process played a relevant role, as the higher temperature value had a significantly better effect on the amount of synthesised LBA. Moreover, the addition of the redox mediators substantially increased the quantity of lactobionic acid—twice in the case of DCPIP and more than three times in the system with ABTS. The maximum conversion efficiencies over the initial substrate concentration were approximately 24% LBA at 30 °C (LBA concentration 12 mM) and 43% LBA at 50 °C (21.28 mM) when ABTS, PlCDH, CuLAC, and lactose were used as substrates. The maximum conversion rate of disaccharide to LBA was about 19% at 30 °C (9.69 mM) and 26% at 50 °C (12.97 mM), where DCPIP, PlCDH, CuLAC, and lactose were the elements of the reaction mixture.

It has been shown that an increasing number of scientists synthesise lactobionic acid with enzymatic methods. The efficiency of lactobionic acid synthesis in the present study is up to about 20 times higher than in our previous study. In this experiment, we were able to synthesise 21 mM sLBA, whereas previously we obtained a maximum of 1 mM LBA using PlCDH as the enzyme. Compared with our previous study, there was clearly an upward trend in the LBA synthesised at present, which allowed us to analyse this compound for properties that are valuable from a biotechnological and industrial point of view [17,19,44].

However, a number of research teams have focused on improving the LBA synthesis process; therefore, it is possible to analyse the effect of various factors on the amount of synthesised lactobionic acid. Over the last few years, enzymatic systems for the production of lactobionic acid have evolved. Reaction mediators have started to be used, which have significantly improved the efficiency of the process. At the beginning of the 21st century, Baminger et al. presented results in which complete conversion of lactose to LBA in the presence of the fungal enzyme CDH from *Sclerotium rolfsii*, lactase from *Trametes pubescens*, and the redox mediator ABTS was possible in 150 min [41]. Furthermore, Van Hecke et al. also addressed the synthesis of lactobionic acid using CDH, LAC, and ABTS; however, they used a 4-fold excess of LAC relative to CDH. Their results show a conversion of lactose into LBA of up to 97% [46]. It is also worth mentioning the study conducted by Ludwig et al., which checked two types of redox mediators—ABTS and DCPIP. Their results show an evidently faster rate of LBA production in the system with ABTS. We also note that ABTS appeared to be a better mediator. The amount of lactobionic acid produced at 50 °C was almost twice as high in the reaction system with ABTS compared with the system with DCPIP [40].

In our study, we focused on fungal enzymes that can be used for the synthesis of lactobionic acid; however, bacterial producers are also very commonly used. Some studies show that the majority of LBA syntheses in laboratory conditions are still based on the use of bacterial enzymes [47,48]. To confirm the presence of lactobionic acid, we additionally used the TLC method. Based on a study conducted by Kiryu et al., we created a system that allowed us to visualise spots corresponding to LBA. Our results were in agreement with the lactobionic acid visualisation reported by these authors [42].

### 3.2. Data Analysis for FTIR Spectra

**Figure 7:**
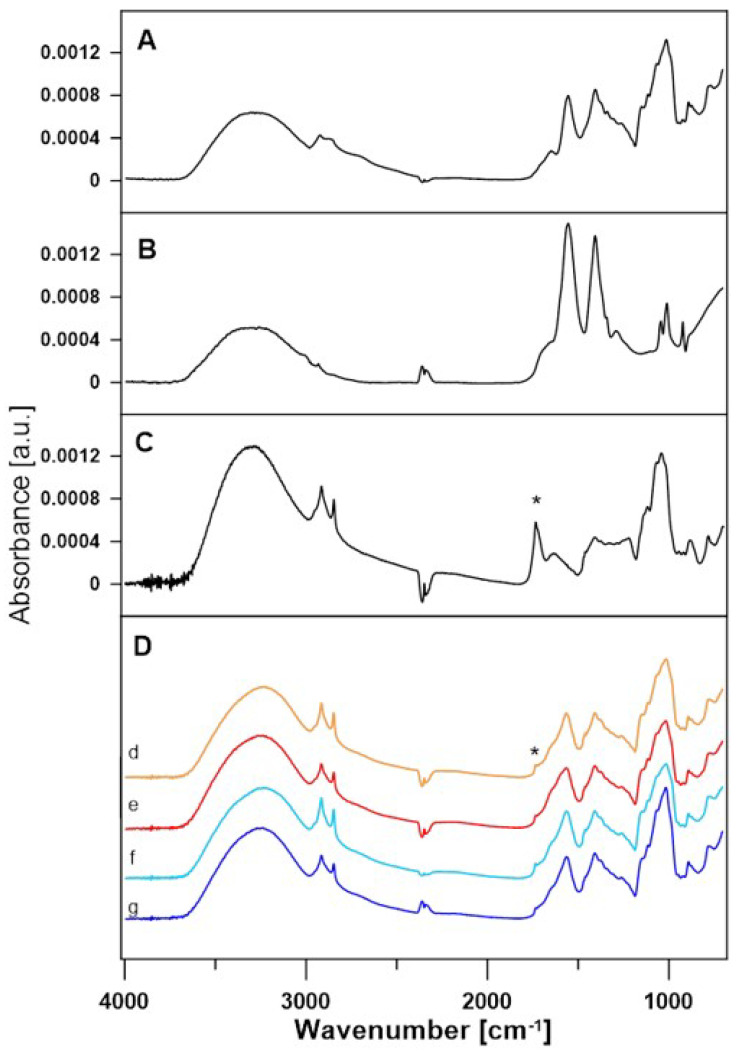
FTIR spectra of lactose (**A**), acetate buffer (**B**) and lactobionic acid (**C**). (**D**) Comparison of FTIR spectra of A30 (d), A50 (e), D30 (f), and D50 (g) samples after enzymatic reaction. An asterisk marks 1735 cm^−1^ band of the C=O_acid_ bond.

After the enzymatic reaction, all samples contained lactose, acetate buffer, and mediators and were assumed to contain lactobionic acid. FTIR experiments were performed to confirm that the reaction was successful. The comparison of the FTIR spectra of the film of the lactose solution in acetate buffer, the acetate buffer spectra, and the spectra of the film of the lactobionic acid solution revealed the presence of the same bands, which are related to the chemical structure of LBA and lactose molecules. A difference that indicates the presence of LBA in the samples is the 1735 cm^−1^ band (marked with an asterisk), which corresponds to the stretching of the C=O_acid_ bond present in the LBA spectrum and is absent in the spectra of the other compounds (Figure 7) [49,50]. The spectra of samples containing LBA formed in the enzymatic reaction with ABTS at 30 °C (A30), ABTS at 50 °C (A50), DCPIP at 30 °C (D30), and DCPIP at 50 °C (D50) are shown in Figure 7.

**Figure 8:**
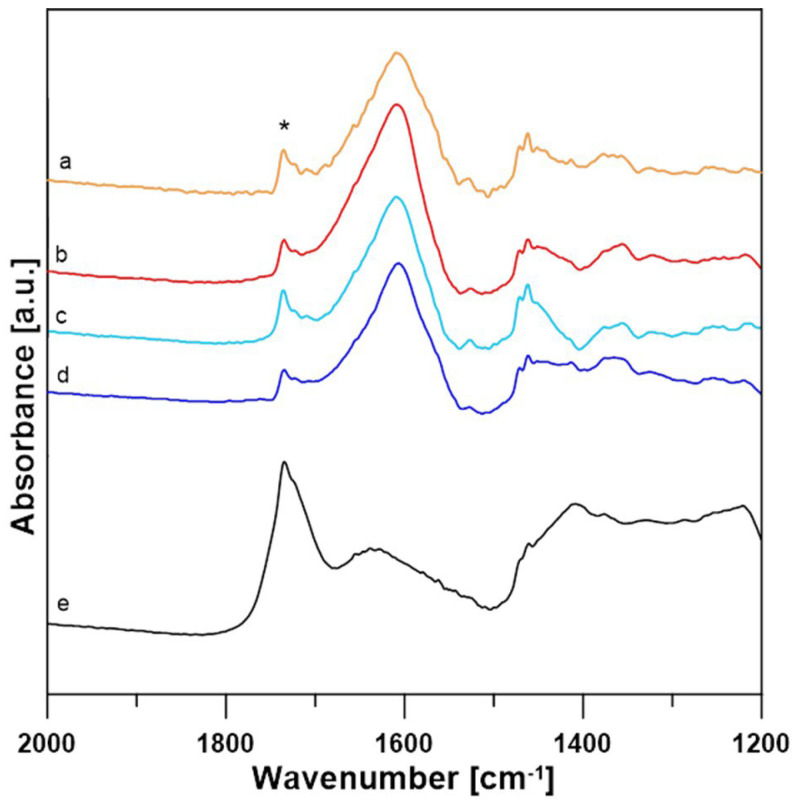
Comparison of sample-lactose differential spectra of A30 (a), A50 (b), D30 (c), D50 (d) and pure LBA (e). An asterisk * marks 1735 cm^−1^ band of the C=O_acid_ bond.

As can be seen, each of these spectra correspond to acetate buffer and lactose. A 1735 cm^−1^ band is also present, which suggests that lactobionic acid is present in all of the samples. For better demonstration, the lactose spectrum was subtracted from the spectra of all samples. Differential spectra are shown in Figure 8.

It has been shown that the 1735 cm^−1^ stretching C=O bond can be used as an indicator of the presence of LBA in these samples, and its presence in the differential spectra indicates that the samples contain traces of lactobionic acid.

### 3.3. Antioxidant Properties of LBA

**Figure 9:**
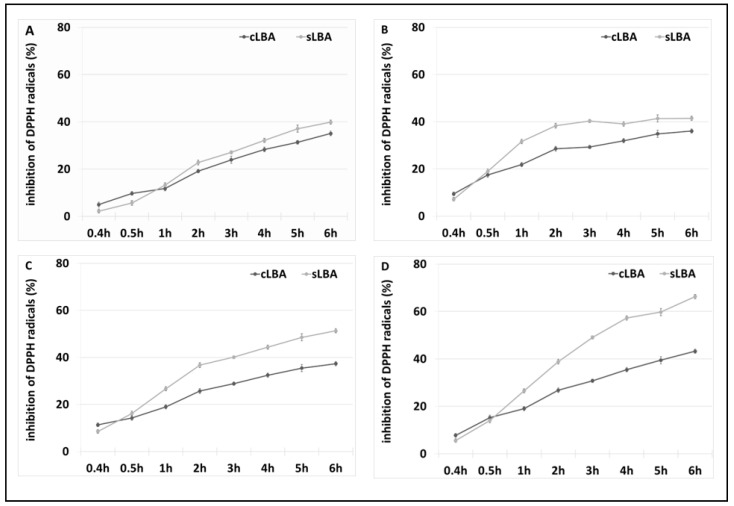
Results of the 2,2-diphenylpicrylhydrazyl (DPPH) antioxidant test. (**A**) Antioxidant properties of a reaction mixture containing 12 mM LBA derived from the oxidation of lactose in the presence of ABTS at 30 °C and commercial LBA with the same concentration, (**B**) 21 mM LBA derived from the oxidation of lactose in the presence of ABTS at 50 °C and commercial LBA with the same concentration, (**C**) 10 mM LBA extracted via lactose oxidation in the presence of DCPIP at 30 °C and commercial LBA with the same concentration, and (**D**) 13 mM LBA extracted via lactose oxidation in the presence of DCPIP at 50 °C and commercial LBA with the same concentration. Error bars represent the standard deviation of the three experiments.

The antioxidant properties of the LBA contained in the reaction mixture were assessed using the DPPH antioxidant activity assay. A commercial LBA (cLBA) reagent was used as a control at the same concentrations as in the samples tested. The results are shown in the figure below (Figure 9).

As shown above, the mixture of lactobionic acid synthesised via lactose oxidation exhibited antioxidant properties and, compared with the pure commercial reagent, the compound obtained had a higher ability to absorb DPPH free radicals. The mixture with 13 mM LBA synthesised at 50 °C in the presence of the redox mediator DCPIP showed the best antioxidant properties. After 6 h of incubation, sLBA exhibited a free radical uptake of 60%, where commercial LBA showed 40% less antioxidant capacity after this time. The formulations prepared using DCPIP as a redox mediator had better antioxidant properties than those with ABTS.

We also examined the free radical scavenging ability of DCPIP and lactose (controls). These compounds did not show antioxidant properties. This confirms that the produced lactobionic acid in the samples with DCPIP as a mediator, has antioxidant activity.

The free radical scavenging capacity of lactobionic acid is increasingly being used in various industries. Literature data report that LBA has antioxidant properties; hence, we decided to determine the antioxidant properties of a reaction mixture containing sLBA [31,51,52].

Research conducted by Cardoso suggests using the method with ABTS to determine the antioxidant properties of formulations analysed in the present study. However, due to the presence of ABTS in our samples, we ruled this method out and decided to use the method with DPPH to analyse sLBA [31]. In our study, 13 mM sLBA extracted in the presence of DCPIP at 50 °C after 6 h had a free radical scavenging capacity of approximately 70%. Our results are much higher than those reported in the literature. As reported by Tasic-Costov, the maximum antioxidant activity of LBA was 60% [52].

Most importantly, Cardoso et al. did not observe a correlation between the LBA concentration and an increase in antioxidant properties, and neither did we. This may indicate a lack of correlation between the LBA concentration and the free radical scavenging efficiency of the compound [31]. It is well known that lactobionic acid has recently become increasingly popular due to its unique properties, including its antioxidant properties. In the food industry, this compound prolongs the shelf life of products [31,53]. Furthermore, in the cosmetics industry, LBA is used as an additive in creams to delay the skin ageing process [54].

### 3.4. Antibacterial Properties of LBA

The antibacterial properties of sLBA were determined using the microdilution method evaluating the inhibition of bacterial growth expressed as a percentage and with the TTC method assessing microbial viability. The effect of LBA on microorganisms was determined by the growth of two strains of Gram-negative bacteria (*E. coli* ATCC 25922 and *P. aeruginosa* ATCC 27853) and two strains of Gram-positive bacteria (*S. aureus* ATCC 25923 and *S. epidermidis* ATCC 14990). The results of the inhibition of bacterial growth under the influence of the examined reaction mixtures are presented in Table 2.

LBA synthesised via lactose oxidation showed better antibacterial properties against Gram-negative bacteria. In the case of Gram-positive bacteria, LBA produced in the presence of DCPIP exerted substantially higher effects (even 60% better results) than the acid synthesised in the reaction in the presence of ABTS. The lowest concentration of sLBA (9.69 mM) appeared to have the best effect on the inhibition of *S. epidermidis* growth. This may be valuable information in terms of the economic use of sLBA in the biotechnology industry. The comparison of the pure commercial reagent with the effect of the acid extracted in the present study revealed evident differences. Our sample had a major effect on the viability of *P. aeruginosa* and *E. coli* in all reaction conditions.

The TTC method facilitated determination of the number of metabolically active cells. These results are presented in Table 3.

In all the variants tested, LBA markedly affected the bacterial biofilm-forming capacity. The lowest effect (50%) was observed in samples synthesised with the use of ABTS. The impairment in biofilm formation caused by the commercial lactobionic acid formulation in the Gram-negative bacteria was scarcely noticeable. In turn, our mixture strongly reduced the metabolic activity of these bacteria. The biofilm-forming ability of *S. aureus* was maintained at 25%, except for the preparation with ABTS incubated at 50 °C, as almost half of the bacteria retained metabolic activity in this case. More importantly, *S. epidermidis* retained 50% metabolic activity after the use of the mixture with ABTS, whereas the acid produced in the presence of DCPIP allowed only 40% of the bacteria to form a biofilm. Our study shows that lactobionic acid has a profound effect on the metabolic activity of microorganisms.

*Staphylococcus aureus* is a Gram-positive microorganism and a major bacterial pathogen of humans; therefore, research on the limitation of the growth of this microorganism is of great interest to scientists. Cao and co-workers studied the effects of LBA on *S. aureus* bacteria. Their research shows that LBA had significant antibacterial activity—lactobionic acid caused alkaline phosphatase and nucleotide leakage into the culture medium [55,56]. Similarly, Hou et al. focused on an assessment of the effect of LBA on *S. aureus*. They analysed the mechanism of action of LBA towards teichoic acid present in the bacterial cell wall. As shown in their study, lactobionic acid can be successfully used as an anti-biofilm agent [57]. Similarly, Kang et al. investigated the effects of LBA on methicillin-resistant *S. aureus* (MRSA N315). Their study confirms the results presented by other research teams: the antibacterial effect of LBA causes cell wall damage and loss of cell membrane integrity [58].

It is worth mentioning that other research teams investigated the effects of LBA on *E. coli*. Their results also confirm the antibacterial activity of LBA; however, the main focus of their research was the use of LBA in the context of food products [31,59]. A study conducted by Coroli et al. showed the effect of LBA on Gram-positive and Gram-negative bacteria. In both cases, LBA exhibited antibacterial activity, with a stronger effect against Gram-negative bacteria. The results of our study, i.e., an inhibition level of about 90% for *E. coli* and about 60% for *S. epidermidis*, were comparable to those reported by Coroli et al., [60]. Kang et al. studied the effect of LBA on *Pseudomonas fluorescens* and the results of the antibacterial activity show that *P. fluorescens* was more susceptible to LBA at the lowest minimum bactericidal concentration [61].

Recent studies have shown that the antibacterial effect of LBA is still being explored by researchers. Zhang et al. studied the effects of LBA on Gram-negative bacterial pathogens of seafood. Their results confirm the bacteria-killing effect of lactobionic acid on *Shewanella baltica* and *Shewanella putrefaciens*. LBA can damage the integrity of the cell membrane and cause protein leakage [62].

## 4. Conclusions

The present study has clearly shown that lactobionic acid can be obtained via synthesis using a multi-enzymatic system. In this study, fungal enzymes extracted from white wood rot fungi (C. unicolor and P. lindtneri) were used for the first time. Lactobionic acid was synthesised in all the enzymatic systems tested. The addition of redox mediators (DCPIP or ABTS) significantly increased the amount of LBA formed, which strongly affected the efficiency of the process. Moreover, a key role was also played by temperature, as the higher value (50 °C) increased the amount of produced LBA. However, we observed differences between the compounds obtained in the different enzymatic conditions. The synthesised lactobionic acid showed varied antioxidant and antibacterial properties. LBA affected the viability of all tested bacterial strains, although the effect was stronger against Gram-negative bacteria. The results show the potential application of LBA formed in multi-enzymatic systems composed of enzymes from white rot fungi in many industries, e.g., in the food industry.

## Figures and Tables

**Table 1:** Amounts of lactobionic acid formed by enzymatic synthesis after 24 h of incubation at 30 °C and 50 °C.

Enzymatic System	Redox Mediator	Temperature	Lactobionic Acid Concentration [mM]	Conversion Efficiencies [%]
PlCDH CuLAC Lactose	ABTS	30 °C	12.00	24.00
		50 °C	21.28	42.56
PlCDH CuLAC Lactose	DCPIP	30 °C	9.69	19.38
		50 °C	12.97	25.94

**Table 2:** Rate of inhibition of the growth of *S. aureus*, *S. epidermidis*, *E. coli*, and *P. aeruginosa* bacteria from a mixture containing lactobionic acid obtained via lactose oxidation in the presence of redox mediators (ABTS or DCPIP).

Enzymatic System/ Bioactive Substances	% Bacterial Growth Inhibition			
	*S. aureus* ATCC 25923 MIC (cLBA) = 1 mg/mL	*S. epidermidis* ATCC 14990 MIC (cLBA) = 2 mg/mL	*E. coli* ATCC 25922 MIC (cLBA) = 4 mg/mL	*P. aeruginosa* ATCC 27853 MIC (cLBA) = 9 mg/mL
PlCDH, CuLAC, Lactose ABTS, T = 30 °C	45.3 ± 0.8	1.3 ± 0.4	89.2 ± 0.4	100.0 ± 0.2
PlCDH, CuLAC, Lactose ABTS, T = 50 °C	13.8 ± 0.5	0.0 ± 0.0	72.9 ± 0.8	81.1 ± 0.7
PlCDH, CuLAC, Lactose DCPIP, T = 30 °C	41.5 ± 0.3	67.4 ± 0.5	92.6 ± 0.7	100.0 ± 0.3
PlCDH, CuLAC, Lactose DCPIP, T = 50 °C	42.4 ± 0.5	44.6 ± 0.4	92.0 ± 0.8	100.0 ± 0.3

**Table 3:** Percentage of metabolically active cells of *S. aureus*, *S. epidermidis*, *E. coli*, and *P. aeruginosa* bacteria from a mixture containing lactobionic acid obtained via lactose oxidation in the presence of redox mediators (ABTS or DCPIP).

Enzymatic System/ Bioactive Substances	% of Metabolically Active Cells			
	*S. aureus* ATCC 25923	*S. epidermidis* ATCC 14990	*E. coli* ATCC 25922	*P. aeruginosa* ATCC 27853
PlCDH, CuLAC, Lactose ABTS, T = 30 °C	27.1 ± 0.6	53.9 ± 0.5	12.6 ± 0.3	14.2 ± 0.5
PlCDH, CuLAC, Lactose ABTS, T = 50 °C	45.4 ± 0.3	52.5 ± 0.3	25.8 ± 0.5	24.7 ± 0.6
PlCDH, CuLAC, Lactose DCPIP, T = 30 °C	25.6 ± 0.7	41.4 ± 0.6	12.5 ± 0.4	12.9 ± 0.8
PlCDH, CuLAC, Lactose DCPIP, T = 50 °C	26.0 ± 0.8	38.0 ± 0.4	12.6 ± 0.3	12.6 ± 0.4

## Data Availability

No new data were created or analyzed in this study. Data sharing is not applicable to this article.
